# Prevalence of phenotypic multi-drug resistant *Klebsiella* species recovered from different human specimens in Ethiopia: A systematic review and meta-analysis

**DOI:** 10.1371/journal.pone.0297407

**Published:** 2024-02-09

**Authors:** Biniyam Kijineh, Tsegaye Alemeyhu, Mulugeta Mengistu, Musa Mohammed Ali

**Affiliations:** 1 Department of Medical Laboratory Science, Wachemo University College of Medicine and Health Sciences, Hossana, Ethiopia; 2 School of Medical Laboratory Science, Hawassa University College of Medicine and Health Sciences, Hawassa, Ethiopia; Wollo University, ETHIOPIA

## Abstract

**Background:**

Multidrug-resistant (MDR) *Klebsiella* species are among public health important bacteria that cause infections difficult to treat with available antimicrobial agents. Infections with *Klebsiella* lead to high morbidity and mortality in developing countries particularly in patients admitted to the intensive care unit. This systematic review and meta-analysis aimed to determine the pooled prevalence of MDR *Klebsiella* species from different human specimens using studies conducted in Ethiopia from 2018−2022.

**Methods:**

We have systematically searched online databases such as PubMed/Medline, Google Scholar, Hinari, African journals online, Web of Science, Cochrane, and grey literature (Addis Ababa University and Hawassa University) to identify studies reporting the proportion of MDR *Klebsiella* species in Ethiopia. Published articles were selected based on the Preferred Reporting Item of Systematic Review and Meta-analysis (PRISMA). R-Studio version 4.2.3 was used to conduct pooled prevalence, heterogeneity test, and publication bias. A binary random effect model was used to determine the pooled prevalence. Heterogeneity was checked with the inconsistency index (I^2^). Publication bias was checked with a funnel plot and Egger test. Sensitivity analysis was conducted with leave-one-out analysis. Joanna Briggs Institute’s critical appraisal tool for prevalence studies was used to check the quality of each article.

**Results:**

In this systematic review and meta-analysis, 40 articles were included in which 12,239 human specimens were examined. Out of the total specimens examined, 721 *Klebsiella* species were isolated and 545 isolates were reported to be MDR *Klebsiella* species. The prevalence of MDR *Klebsiella* species ranged from 7.3%-100% whereas the pooled prevalence of MDR *Klebsiella* species was 72% (95% CI: 63 − 82%, *I*^*2*^ = 95%). Sub-group analysis based on region revealed the highest prevalence of MDR from Addis Ababa (97%) and the least from the Somali region (33%); whereas sub-group analysis based on the specimen type indicated the highest prevalence was from blood culture specimens 96% and the least was from other specimens (ear and vaginal discharge, and stool) (51%).

**Conclusion:**

Our finding indicated a high prevalence of MDR *Klebsiella* species found in different human specimens. The prevalence of MDR *Klebsiella* varies across regions in Ethiopia, age, the type of specimens, source and site of infection. Therefore, integrated action should be taken to reduce the prevalence of MDR *Klebsiella* species in regional states and focus on clinical features. Effective infection and prevention control should be applied to reduce the transmission within and outside health care settings.

## Introduction

*Klebsiella* is a member of the Enterobacteriaceae family and is naturally present in the gastrointestinal tract microbiome of healthy humans and animals [1]. This common pathogen causes hospital-acquired surgical wound infections, digestive tract infections, community-onset infections, and nosocomial infection outbreaks [2].

Genus *Klebsiella* includes a variety of species that cause human such as *Klebsiella pneumoniae*, *Klebsiella ozaenae*, *Klebsiella rhinoscleromatis*, *Klebsiella oxytoca and Klebsiella aerogenes* [3]. The two main pathotypes of *Klebsiella* species, the multidrug-resistant (MDR) and hyper-virulent (hv) clones, account for a significant fraction of infections [4]. The two branches’ strains were regarded as non-overlapping since their respective genetic backgrounds differed [4]. Nevertheless, it has been shown that *Klebsiella* species. can obtain genetic elements and mutations that confer virulence traits and/or antibiotic resistance, which ultimately leads to the emergence of convergent clones known as multidrug-resistant and hyper-virulent (MDR-hv) *Klebsiella* spp [5, 6]. MDR-hv *Klebsiella* species exhibit dual hyper-virulence and antibiotic resistance, and they are believed to be evolving further to generate phenotypically distinct strains [6, 7]. Numerous reports from various continents worldwide have documented a wide range of MDR-hv strains of *Klebsiella* species that have evolved through various mechanisms [8]. MDR-hv *Klebsiella* species have become real superbugs that pose major threats to public health due to the rise in severe infections and the growing lack of effective treatments [9].

In the past decade, antibiotic resistance has become a major global public health concern. Antimicrobial resistance (AMR)-related infections caused 700,000 deaths worldwide each year; by 2050, this number is predicted to increase to 10 million worldwide and 4.2 million in Africa [10]. According to reports, there is a 70% global rate of antibiotic resistance for *Klebsiella*, and the incidence of infection-related mortality ranges from 40% to 70% [4]. In recent years, there has been a growing concern for global public health regarding carbapenem-resistant *Klebsiella* (CRK) and multiple-drug-resistant *Klebsiella* (MDRK). *Klebsiella* species was listed as one of the eight drug-resistant microorganisms in a 2017 WHO report. The advent of multidrug-resistant *Klebsiella* species and limited therapeutic options for infections caused by multidrug-resistant *Klebsiella* species has made treating infections caused by these species more difficult at the moment [11, 12].

Even though there is a clear correlation between colonization and infection, it is unknown what risk factors colonized patients have for infection. It is most likely determined by a combination of bacterial and patient factors whether a patient becomes infected with *Klebsiella*. In a population-level analysis lacking colonization assessment, *Klebsiella* bacteremia was linked to advanced age, male sex, dialysis, chronic liver disease, solid organ transplant, and cancer [13]. Advanced age is linked to the colonization of *Klebsiella* [14]. *Klebsiella* genes and patient characteristics linked to infection with *Klebsiella* as opposed to asymptomatic colonization [15].

Due to their multidrug resistance, *Klebsiella* species is now considered an urgent threat to human health; however, the MDR profile varies amongst countries, even when it comes to the widespread use and misuse of antimicrobial agents [16]. The epidemiology and drug susceptibility pattern of *Klebsiella* species have been studied in various regions of Ethiopia at different times, but the pooled prevalence of MDR *Klebsiella* species in Ethiopia has not been reported. This systematic review and meta-analysis aimed to determine the pooled prevalence of MDR *Klebsiella* species in Ethiopia based on regional states, specimen types, age, source and site of infections.

## Methods

### Literature search strategy

We have systematically searched online databases such as PubMed/Medline, Google Scholar, Hinari, African journals online, Web of Science, Cochrane, and grey literature (Addis Ababa University and Hawassa University) to identify studies reporting the proportion of multi-drug resistant *Klebsiella* species in Ethiopia. Search terms were grouped into four queries (prevalence, antimicrobial susceptibility related term, *Klebsiella spp*, and Ethiopia). The Boolean operator, ‘or’ (within a query) ‘or’ (between antibacterial resistance terms), Resistance search terms include “Antibiotic-resistant *Klebsiella*”, “Antibiotic susceptible *Klebsiella*”, “*Klebsiella* antibiotic sensitivity”, “Antimicrobial susceptibility of *Klebsiella*”, “Antimicrobial sensitivity of *Klebsiella*”, “Antimicrobial resistance of *Klebsiella*”, “Antibacterial resistance of *Klebsiella*”, “Resistance of *Klebsiella*”, “Drug resistance of *Klebsiella*”. Studies published from January 1, 2018, to March 3, 2023, were included. We followed the Preferred Reporting Item of Systematic Review and Meta-analysis (PRISMA) to select the articles [17] (S1 Table).

### Data extraction

The data were extracted by three researchers (BK, TA, and MMA) using a standardized and pretested format on April 1–7, 2023. The data abstraction format included the first author, study site, specimen, lab method, sample size, the number of *Klebsiella* species isolated and MDR profile of *Klebsiella* species. Any disagreement on the study to be included/excluded during data extraction between researchers was handled through discussion.

### Quality assessment

Before considering the articles for systematic review and meta-analysis, their quality was checked using nine criteria mentioned in the Joanna Briggs Institute (JBI) critical appraisal tool for prevalence studies [18]. The checklist includes the following questions: Was the sample frame appropriate to address the target population? Were study participants sampled appropriately? Was the sample size adequate? Were the study subjects and the setting described in detail? Was the data analysis conducted with sufficient coverage of the identified sample? Were valid methods used for the identification of the condition? Was the condition measured in a standard, reliable way for all participants? Was there an appropriate statistical analysis? Was the response rate adequate, and if not, was the low response rate managed appropriately? Articles that scored more than 50% were considered good quality articles and included in the systematic review and meta-analysis [19] (**S2 Table**).

### Eligibility criteria

Articles with a cross-sectional study design, original studies written in English with full-text access, reported MDR *Klebsiella* species, studies conducted only on human specimens, and studies considering both symptomatic and asymptomatic sources of the specimen were included. Studies with quality scores of less than 50% were excluded from the study.

### The outcome of the study

The outcome of this study was the pooled prevalence of MDR *Klebsiella* species recovered from diverse clinical specimens based on the Regional State of Ethiopia, study participants’ age, specimen types, source, and site of infection.

### Objective of the study

The systematic review and meta-analysis aimed to determine the pooled prevalence of MDR *Klebsiella* species recovered from diverse human specimens in Ethiopia using studies conducted within the time of 2018–2023.

### Statistical analysis

Data analysis was conducted using R-Studio version 4.2.30 to determine the pooled prevalence, publication bias, and heterogeneity test. A binary random-effects model was used to determine the effect size for the proportion of MDR *Klebsiella*. Subgroup analysis was conducted based on the regional state and specimen types used by the studies. The heterogeneity of the articles was determined using the inconsistency index (I ^2^). Publication bias among the studies was tested with a funnel plot and Eggers, and a *p*-value of < 0.05 was considered statistically significant.

## Results

### Search results

**Fig 1:**
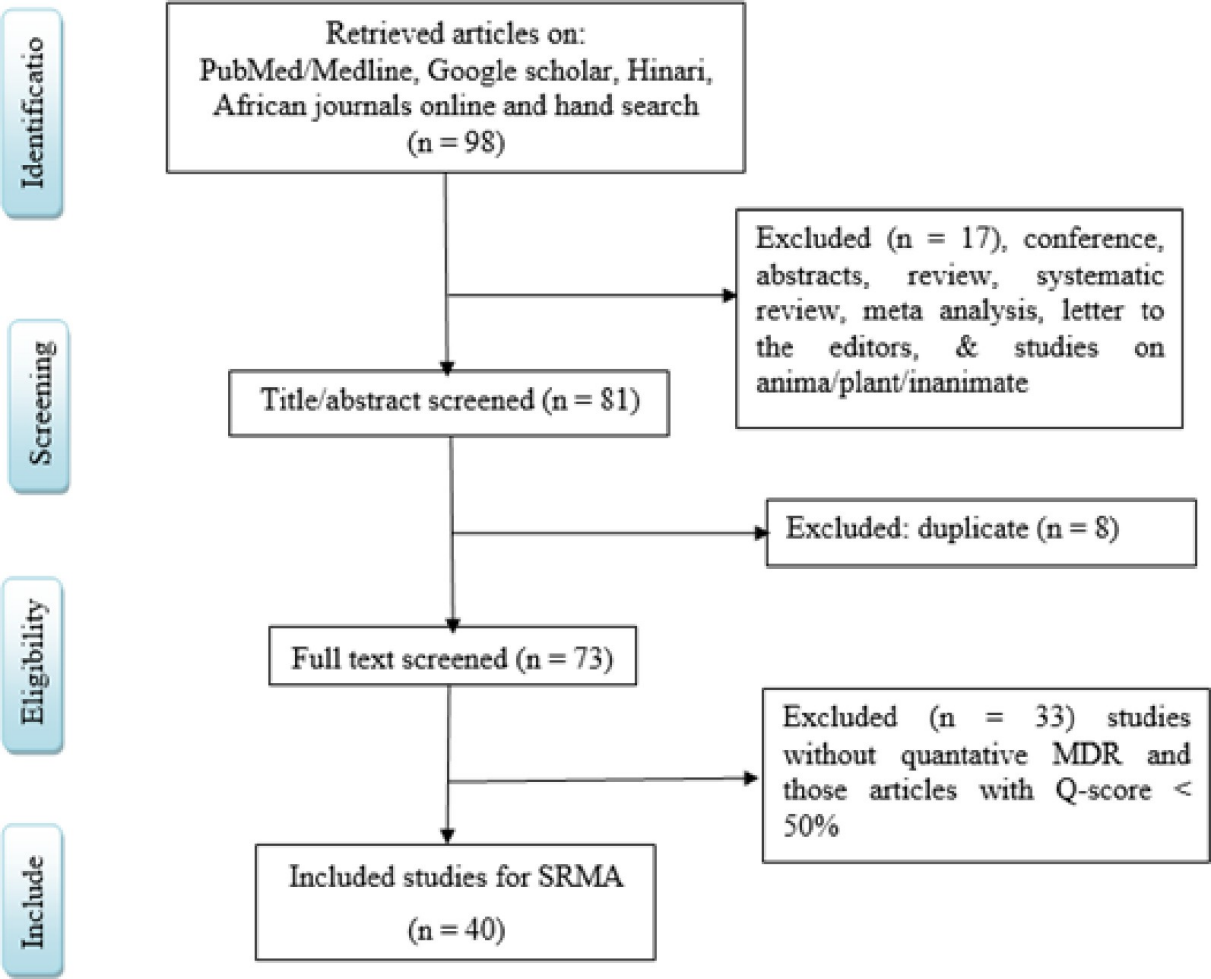
A PRISMA diagram that shows the flow of article selection study characteristics.

After a thorough search, 98 articles were retrieved, of these, 17 belonged to conference papers, systematic reviews, meta-analyses, letters to the editors, and studies on animal/plan and inanimate objects; they were excluded from the study. After the screening of the title and abstract, 8 articles were duplicates and excluded from the study. Further, 73 articles were screened for full paper, of these 33 articles were excluded from the study because they did not report a quantitative MDR *Klebsiella*, and those articles with Q—score < 50%. Based on this, 10 studies scored 90%, 18 studies scored 80%, 9 studies scored 70%, 2 studies scored 60%, and 1 study scored 50%. Finally, 40 articles were included for both systematic review and meta-analysis (**Fig 1**).

**Fig 2:**
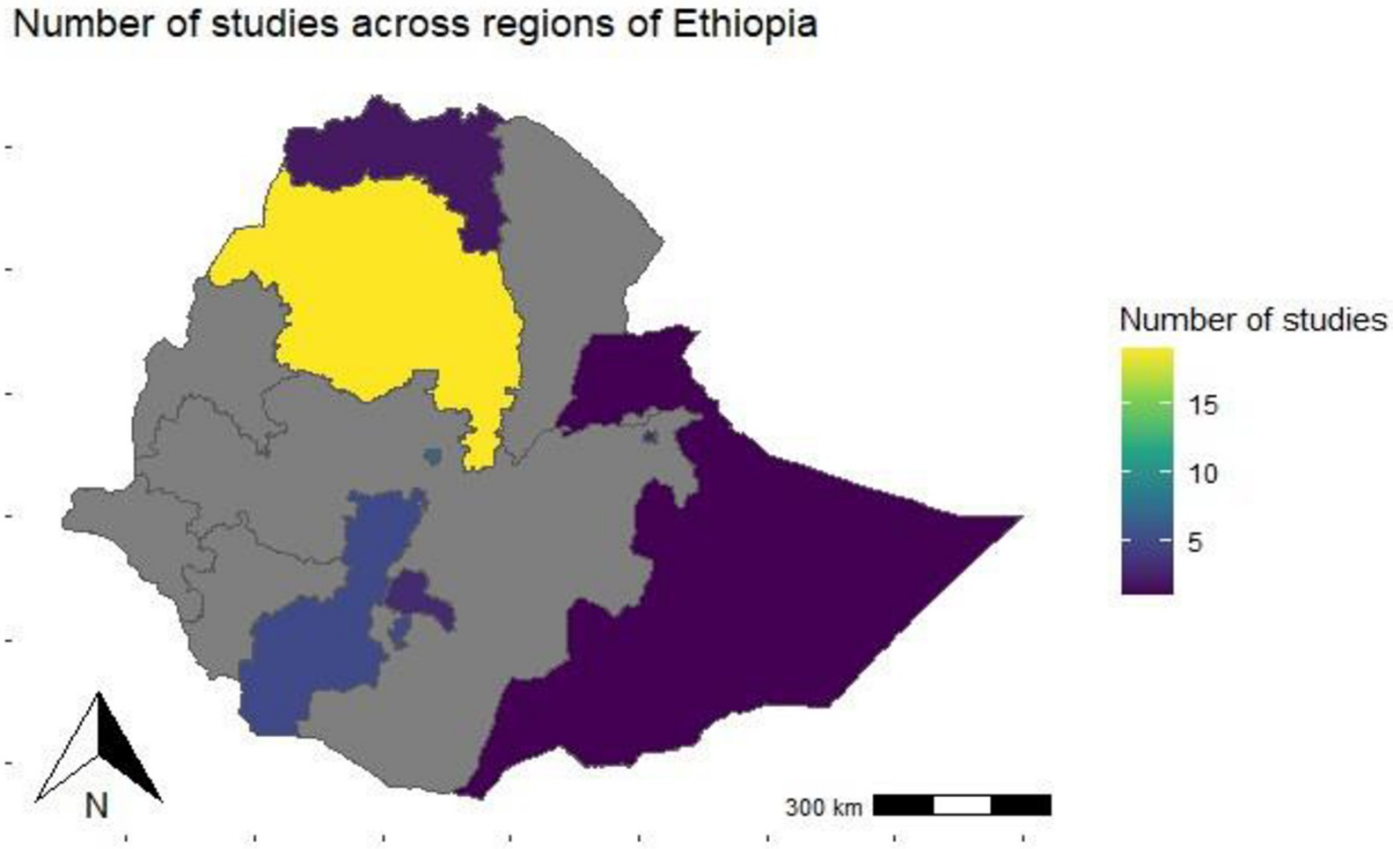
The map that shows the regional states from which the studies included for the study.

Ethiopia is a country of 11 regional states [Afar, Amhara, Benshangual-Gumuz, Gambela, Harari, Oromia, Somali, Southern Nation’s Nationalities, and People’s Region (SNNPR), Tigray, Sidama, and Southwest Ethiopia] and two federal cities [Addis Ababa and Dire Dawa]. Article from 6 regions (Amhara, SNNPR, Sidama, Harari, Tigray, Somali) and one federal city Addis Ababa) met the criteria. A total of 40 articles that were published from 2018–2022 identified for the study, including 19 articles from Amhara region [20–38], 7 articles from Addis Ababa [39–45], 5 articles from SNNPR [46–50], 3 articles from Sidama [51–53], 3 articles from Harari [54–56], 2 articles from Tigray [57, 58] and 1 article from Somali [59] **(Fig 2**).

Concerning articles based on specimen type, most of the articles (21 articles) reported MDR *Klebsiella* from urine [21–23, 25, 27, 30, 33, 38–41, 43, 44, 48–50, 53, 56, 58, 59]. It is followed by seven (7) articles from blood [20, 35, 37, 42, 46, 47, 51], 4 articles from sputum [24, 34, 52, 57], 3 articles from eye swabs [26, 28, 45], 2 articles from body fluid [31, 55], 1 article for each of this specimen stool [32]: vaginal discharge [36] and ear discharge [29]. All the susceptibility testing was conducted with the disk diffusion method. A total of 12,239 patient specimens were analyzed from this, 721 *Klebsiella* were isolated, and of this 545 were reported as MDR (**Table 1**).

**Table 1:** Multi-drug resistant *Klebsiella* species isolated from different human specimens in Ethiopia (2018–2022).

SN	Author	Pub year	Study site	Specimen	Study participants Age	Site of Infections	Source of infection	Sample Size	Total-KLB	MDR-KLB	MDR-KLB (%)	Q-score
1.	Admas *et al* [20]	2020	Amhara	Blood	Adult	BSI	Both	166	7	7	100	8
2.	Adugna [21]	2021	Amhara	Urine	All age group	UTI	Both	422	7	2	28.6	9
3.	Ali *et al* [22]	2018	Amhara	Urine	Reproductive age	AUTI	CAI	358	2	2	100	8
4.	Ameshe [23]	2020	Amhara	Urine	All age group	UTI	Both	385	38	26	68.4	8
5.	Assefa *et al* [24]	2022	Amhara	Sputum	Adult	RTI	CAI	312	39	37	94.9	8
6.	Belete *et al* [25]	2022	Amhara	Urine	Children	UTI	CAI	259	5	5	100	8
7.	Belyhun *et al* [26]	2018	Amhara	Eye swab	All age group	OI	CAI	210	8	5	62.5	5
8.	Girma [27]	2022	Amhara	Urine	All age group	UTI	Both	141	8	3	37.5	6
9.	Haile *et al* [28]	2022	Amhara	Eye swab	All age group	OI	CAI	207	5	1	20	8
10.	Molla *et al* [29]	2019	Amhara	Other	All age group	Other*	CAI	62	10	2	20	6
11.	Oumero *et al* [30]	2022	Amhara	Urine	All age group	UTI	CAI	282	12	6	50	9
12.	Sahle *et al* [31]	2022	Amhara	Body fluid	All age group	MI	HAI	384	58	57	98.3	7
13.	Shenkute *et al* [32]	2022	Amhara	Other	All age group	Other*	HAI	383	102	101	99	7
14.	Tigabu *et al* [33]	2020	Amhara	Urine	All age group	AUTI	CAI	240	7	3	42.9	8
15.	Tilahun *et al*. [34]	2023	Amhara	Sputum	All age group	RTI	CAI	378	46	43	93.5	8
16.	Worku & Tigabu [35]	2022	Amhara	Blood	All age group	BSI	CAI	200	8	3	37.5	7
17.	Yasin *et al* [36]	2022	Amhara	Other	Adult	Other*	CAI	214	7	2	28.6	8
18.	Molla *et al* [37]	2021	Amhara	Blood	Neonate	BSI	CAI	412	57	53	93	7
19.	Fenta *et al* [38]	2020	Amhara	Urine	Children	UTI	CAI	299	7	6	85.7	7
20.	Bizuayehu *et al* [39]	2022	Addis Ababa	Urine	Adult	UTI	HAI	220	9	9	100	7
21.	Bizuwork *et al* [40]	2021	Addis Ababa	Urine	Reproductive age	AUTI	CAI	283	8	8	100	8
22.	Duffa *et al* [41]	2018	Addis Ababa	Urine	Children	UTI	Both	384	17	15	88.2	8
23.	Sherif [42]	2022	Addis Ababa	Blood	Neonate	BSI	Both	400	37	36	97.3	8
24.	Wabe *et al* [43]	2020	Addis Ababa	Urine	Reproductive age	UTI	CAI	290	4	3	75	9
25.	Yenehun *et al* [44]	2022	Addis Ababa	Urine	Adult	UTI	CAI	225	3	3	100	8
26.	Woreta *et al* [45]	2022	Addis Ababa	Eye swab	All age group	OI	CAI	323	6	6	100	8
27.	Ameya *et al* [46]	2022	SNNPR	Blood	Children	BSI	Both	238	4	4	100	9
28.	Birru [47]	2021	SNNPR	Blood	Adult	BSI	Both	225	4	3	75	9
29.	Hantalo *et al* [48]	2022	SNNPR	Urine	Adult	UTI	CAI	217	3	3	100	9
30.	Mitiku *et al* [49]	2022	SNNPR	Urine	Adult	UTI	CAI	422	39	22	56.4	9
31.	Oumer *et al* [50]	2022	SNNPR	Urine	Adult	UTI	HAI	231	9	9	100	7
32.	Alemayehu *et al* [51]	2019	Sidama	Blood	Children	BSI	HAI	939	21	21	100	8
33.	Gebre [52]	2021	Sidama	Sputum	Adult	RTI	CAI	406	36	12	33.3	9
34.	Mechal *et al* [53]	2022	Sidama	Urine	Adult	UTI	CAI	395	14	9	64.3	9
35.	Ejerssa *et al* [54]	2021	Harari	Urine	Reproductive age	UTI	CAI	200	5	3	60	8
36.	Tolera *et al* [55]	2018	Harar	Body fluid	All age group	MI	HAI	394	4	2	50	8
37.	Marami D [56]	2019	Harar	Urine	Adult	UTI	CAI	350	15	4	26.7	9
38.	Adhanom *et al* [57]	2019	Tigray	Sputum	Adult	RTI	Both	252	41	3	7.3	7
39.	Gebremariam *et al* [58]	2019	Tigray	Urine	Adult	UTI	CAI	341	6	5	83.3	8
40.	Negussie *et al* [59]	2018	Somali	Urine	Reproductive age	UTI	CAI	190	3	1	33.3	7
Total								12,239	721	545		314

**Keynote**: Other (vaginal, ear discharge, & stool), Other* (Otitis media, gastrointestinal colonization, and bacterial vaginalis), UTI- urinary tract infection, BSI—bloodstream infection, OM-otitis medium, OI- ocular infection, AUTI- asymptomatic urinary tract infection, RTI-respiratory tract infection, MI-multiple infection, HAI- hospital-acquired infection, and CAI- community-acquired infection.

### Pooled prevalence of MDR *Klebsiella* species

**Fig 3:**
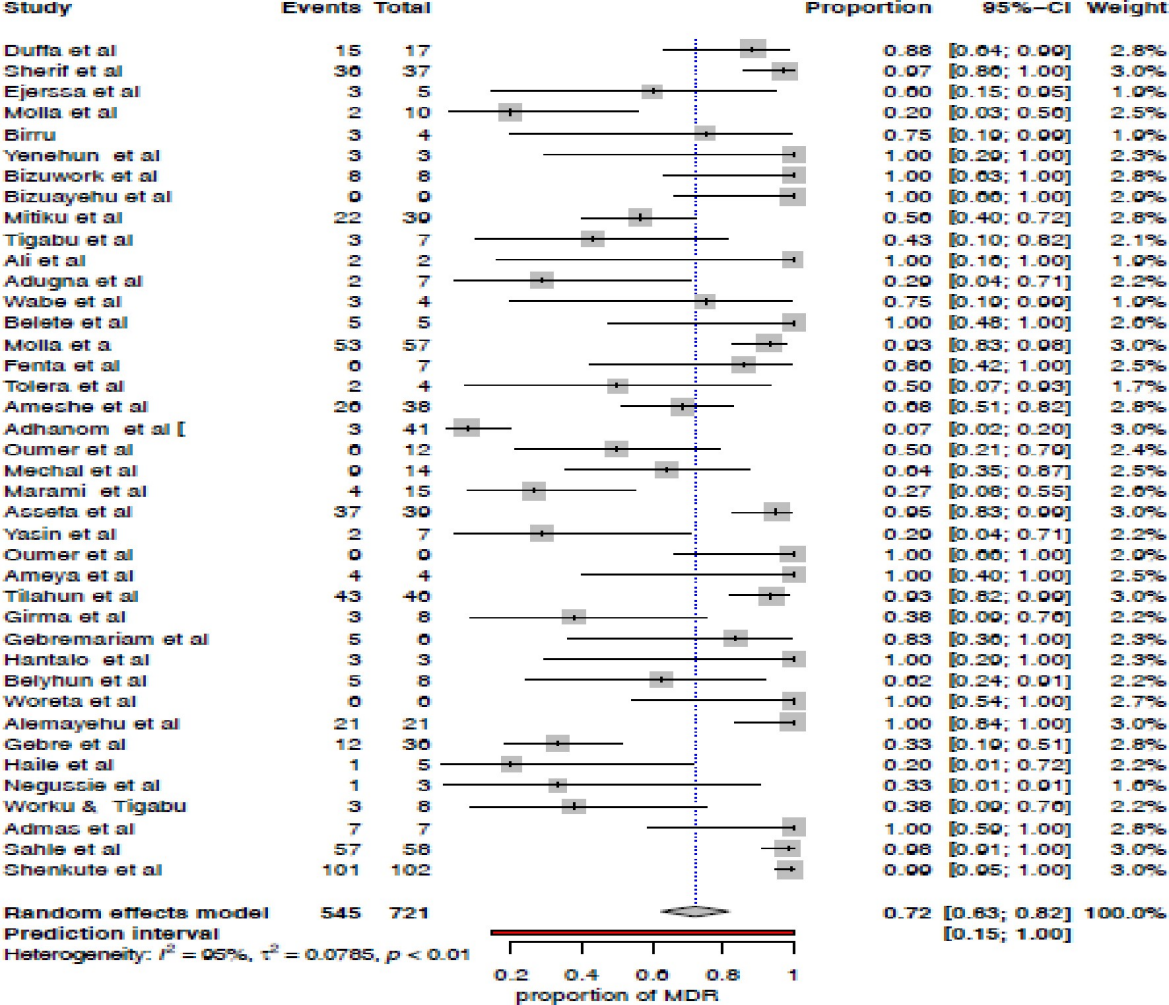
The forest plot that indicates the pooled prevalence of MDR *Klebsiella* species.

A total of 12, 239 patients specimen were analyzed, from these 721 *Klebsiella* species were isolated, and 545 isolates were reported as MDR *Klebsiella* species. The review result indicates the prevalence ranged from 7.3%-100% (**Table 1**). According to meta-analysis, the overall pooled prevalence of MDR *Klebsiella* species in Ethiopia was 72% (63–82%) with high heterogeneity (*I*^*2*^ = 95%, *p* < 0.01) (**Fig 3**).

Because of statistically significant high heterogeneity, we have conducted subgroup analysis based on the regional state, specimen type, source of infection, age of study participants, and site of infection.

### Subgroup analysis

In the subgroup analysis, the prevalence of MDR *Klebsiella* species based on the regions showed that the highest pooled prevalence was from Addis Ababa [97% (95% CI, 93% - 100%), *I*^*2*^ = 0%, *p* = 0.84] and the least were from Somali [33% (95% CI, 1–91%, *I*^*2*^ = NA, *p* = NA. The subgroup analysis based on specimen type the highest was blood culture specimens [96% (92–1.00%), *I*^*2*^ = 61%, *P* = 0.02] and the least was from other specimens (ear & vaginal discharge, and stool) [51% (95%CI, 0–100%), *I*^*2*^ = 96%, *P* < 0.01]. Based on the study participants’ age the highest and the least proportion of MDR was identified in children [97%(95% CI, 91–100%), *I*^*2*^ = 0%, *P* = 0.53] and all age groups [61% (95% CI, 44–78%), *I*^*2*^ = 91%, *P* < 0.01]. The site of infection revealed that BSI had the highest proportion of MDR *Klebsiella* spp. (96% (95% CI, 92–1.00%), *I*^*2*^ = 61%, *P* = 0.02], while other sites of infection (otitis media, gastrointestinal colonization, and bacterial vaginalis) had the lowest rate (51% (95% CI, 00.0–1.00%), *I*^*2*^ = 96%, *P* < 0.01). In the other subgroup analysis based on the source of infection, healthcare-associated infections (HAI) were found to have the highest proportion [99% (95% CI, 97–1.00%), *I*^*2*^ = 0%, *P* = 0.053], which were followed by community-acquired infections (CAI) [68% (95% CI, 56–88%, *I*^*2*^ = 87%, *P* < 0.01] and both HAI & CAI (67% [95% CI, 44–91%, *I*^*2*^ = 98, *P* < 0.01]) (Table 2).

**Table 2:** Subgroup analysis based on regional state, specimen types, study participants’ age, site of infection and source of infection.

Subgroup	No of studies	MDR [95%CI]	Heterogeneity	
			*P*-value	*I*^*2*^ (%)
**Regional state**				
Addis Ababa	7	0.97 [0.93,1.00]	= 0.84	0
Harari	3	0.39[0.16, 0.62]	= 0.34	8
Amhara	19	0.69[0.55,0.83]	< 0.01	89
SNNPR	5	0.69[0.66,1.00]	< 0.01	80
Tigray	2	0.44[0.00,1.00]	< 0.01	96
Sidama	3	0.66[0.27,1.00]	< 0.01	97
**Specimen type**				
Urine	21	0.73[0.63,0.85]	< 0.01	79
Blood	7	0.96[0.92,1.00]	= 0.02	61
Body fluid	2	0.81[0.35,1.00]	= 0.05	73
Sputum	4	0.57[0.14,1.00]	< 0.01	97
Eye swab	3	0.63[0.17,1.00]	< 0.01	88
Other	3	0.51[0.00–100]	<0.01	96
**Study participants age**				
All age groups	14	0.61[0.44–0.78]	< 0.01	91
Adult	14	0.69[0.51–0.87]	< 0.01	97
Neonates	2	0.96[0.92–1.00]	= 0.32	0
Children	5	0.97[0.91–1.00]	= 0.58	0
Reproductive age women	5	0.80[0.57–1.00]	= 0.07	53
**Site of Infection**				
UTI	18	0.72[0.60–0.84]	< 0.01	79
BSI	7	0.96[0.92–1.00]	= 02	61
RTI	4	0.57[0.14–1.00]	< 0.01	99
OI	3	0.63[0.17–1.00]	< 0.01	88
ASUTI	3	0.82[0.46–1.00]	= 0.02	75
MI	2	0.81[0.35–1.00]	= 0.03	73
Other*	3	0.51[0.00–1.00]	< 0.01	96
**Source of infection**				
HAI	6	0.99[0.97–1.00]	= 0.53	0
CAI	25	0.68[0.56–0.88]	< 0.01	87
Both HAI & CAI	9	0.67[0.44–0.91]	< 0.01	98

### Meta-regression

Meta-regression was conducted using regional state, types of specimens, study participants’ age, source of infection, site of infection, and publication year to check the source of the heterogeneity but no variables were significantly associated (**Table 3**).

**Table 3:** Shows the meta-regression based on regional states, types of specimens, study participants’ age, source of infection, site of infection and publication year.

Variables	DF	Coefficient	p-value
Regional state	7	11.88	0.11
Types of specimens	7	10.56	0.16
Study participants age	8	7.03	0.53
Source of infection	2	5.19	0.08
Site of infection	8	10.42	0.24
Publication year	1	2.80	0.10

### Publication bias

**Fig 4:**
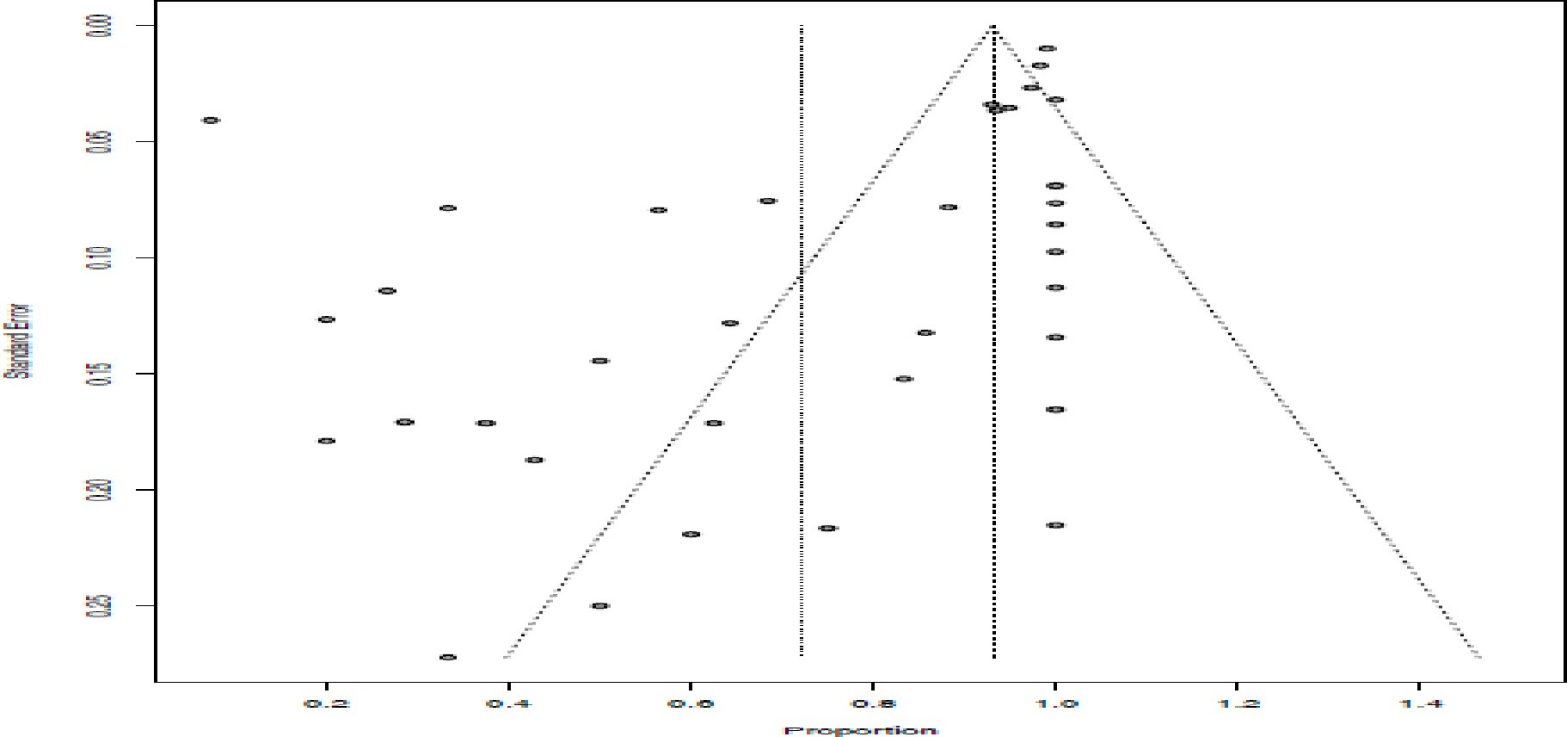
A funnel plot that indicates the publication bias of the studies.

The funnel plot’s asymmetry suggests that there was publication bias, which is corroborated by Egger’s test (*P* < 0.0001) (**Fig 4**).

### Sensitivity analysis

During sensitivity analysis using leave-one-out analysis revealed that the pooled prevalence range of MDR *Klebsiella* remains unchanged (**Table 4**).

**Table 4:** Shows sensitivity analysis with the leave-one-out analysis.

S. No	Study Omitted	MDR [95%CI]	tau^2
1.	Admas *et al* [20]	0.75[0.72; 0.78]	4.11
2.	Adugna [21]	0.74[0.71; 0.78]	3.86
3.	Ali *et al* [22]	0.76[0.72; 0.79]	4.13
4.	Ameshe [23]	0.76 [0.73; 0.79]	3.85
5.	Assefa *et al* [24]	0.76[0.72; 0.79]	4.12
6.	Belete *et al* [25]	0.76[0.72; 0.79]	3.93
7.	Belyhun *et al* [26]	0.75[0.72; 0.78]	3.83
8.	Girma [27]	0.75[0.72; 0.78]	3.82
9.	Haile *et al* [28]	0.77[0.73; 0.80]	4.14
10.	Molla *et al* [29]	0.76[0.73; 0.79]	4.07
11.	Oumero *et al* [30]	0.76 [0.72; 0.79]	3.96
12.	Sahle *et al* [31]	0.76[0.73; 0.79]	3.96
13.	Shenkute *et al* [32]	0.76[0.72; 0.79]	4.12
14.	Tigabu *et al* [33]	0.75[0.72; 0.78]	3.89
15.	Tilahun *et al*. [34]	0.74[0.71; 0.77]	4.06
16.	Worku & Tigabu [35]	0.76[0.72; 0.79]	4.10
17.	Yasin *et al* [36]	0.76[0.73; 0.79]	4.10
18.	Molla *et al* [37]	0.76[0.73; 0.79]	4.17
19.	Fenta *et al* [38]	0.797[0.77; 0.82]	3.44
20.	Bizuayehu *et al* [39]	0.76[0.73; 0.79]	4.11
21.	Bizuwork *et al* [40]	0.76[0.73; 0.79]	4.16
22.	Duffa *et al* [41]	0.77[0.73; 0.80]	3.92
23.	Sherif [42]	0.75[0.71; 0.78]	3.99
24.	Wabe *et al* [43]	0.76[0.73; 0.79]	3.96
25.	Yenehun *et al* [44]	0.75[0.72; 0.78]	3.82
26.	Woreta *et al* [45]	0.76[0.72; 0.79]	3.91
27.	Ameya *et al* [46]	0.74[0.71; 0.78]	4.04
28.	Birru [47]	0.76[0.73; 0.79]	4.03
29.	Hantalo *et al* [48]	0.76[0.72; 0.79]	4.11
30.	Mitiku *et al* [49]	0.76[0.72; 0.79]	3.93
31.	Oumer *et al* [50]	0.76[0.73; 0.79]	4.15
32.	Alemayehu *et al* [51]	0.75 [0.72; 0.78]	3.87
33.	Gebre [52]	0.75[0.72; 0.78]	3.68
34.	Mechal *et al* [53]	0.78[0.75; 0.81]	3.99
35.	Ejerssa *et al* [54]	0.76 [0.73; 0.79]	3.89
36.	Tolera *et al* [55]	0.76[0.73; 0.79]	4.02
37.	Marami D [56]	0.76[0.73; 0.79]	4.03
38.	Adhanom *et al* [57]	0.75[0.72; 0.78]	3.85
39.	Gebremariam *et al* [58]	0.74[0.70; 0.77]	3.75
40.	Negussie *et al* [59]	0.72[0.68; 0.75]	3.59

## Discussion

In hospitalized patients, as well as those in nursing homes and other healthcare facilities, *Klebsiella* species can cause a range of infections, including bloodstream infections, pneumonia, and urinary tract infections more seriously in immunocompromised patients [60]. *Klebsiella* are often resistant to multiple antibiotics. Evidence implicates plasmids as the primary source of the resistance genes. *Klebsiella* species with the ability to produce extended-spectrum beta-lactamases (ESBL) are resistant to virtually all beta-lactam antibiotics, except carbapenems [1]. Developing countries are particularly severely affected by these resistant bacteria if appropriate action is not taken right away. Our study attempted to compile the dispersed data in Ethiopia using a systematic review and meta-analysis because it is one of the nations that has experienced improper antibiotic consumption.

In our finding, the pooled prevalence of MDR *Klebsiella species* was estimated to be 72% (63–82%). Our finding is higher than the study conducted at a global level estimates of MDR *K*. *pneumoniae* 32.8% [61] and Nepal 55% [62]. This difference may be due to their study was conducted on only MDR *K*. *pneumoniae* from hospital-acquired infection isolates and our study included all *Klebsiella* species on both CAI and HAI. Our finding is comparable with systematic review and meta-analysis from Nepal 64% [63] which was conducted on MDR *K*. *pneumoniae* from clinical isolates even if our study is on overall *Klebsiella* species. It is also comparable with a study from Ethiopia 68% [64] which was conducted with one health approach from human, animal, and environmental samples. These similarities indicate MDR *Klebsiella* species is circulating anywhere and fighting AMR with one health approach is much more important nowadays.

Globally, AMR has been documented and has the potential to spread quickly. Along with the usage of antibiotics, there are differences in the distribution of resistance and the number of infections around the world. There is a significant knowledge gap regarding the magnitude of AMR in the world. Particularly the knowledge gap is common in low- and middle-income countries that lack systems to gather data on infections and antibiotic-resistant bacteria [65]. In our systematic review and meta-analysis, a subgroup analysis was conducted based on the regional states, type of specimens, study participants’ age, source of infection (CAI vs HAI), and types of infection as the data is heterogenous (*I*^*2*^ = 95%).

The MDR *Klebsiella* species prevalence varied among the regional states, according to the subgroup analysis. Addis Ababa had the highest pooled prevalence, at 97% (93–100%), while Somali had the lowest, at 33% (1–91%). The number of studies included the nature of study participants, and the sample size could all be contributing factors to this variation. One possible explanation could be variations in access to clean water, hygienic practices, and sanitation for both humans and animals; infection prevention and control measures at homes, hospitals, and farms; availability of reasonably priced vaccines; access to diagnostics and medications; and awareness and knowledge regarding antibiotic resistance and appropriate use of antibiotics, which are the primary risk factors for the development of antibiotic resistance as outlined by the World Health Organisation [66].

The sub-group analysis based on the types of specimens shows that the blood culture had the highest prevalence of MDR Klebsiella spp., at 96% (92–100%), while the other specimen cultures (stool, ear discharge, and vaginal discharge) had the lowest prevalence, at 51% (0.0–100). This can be explained by the fact that the latter specimen came from infections acquired in the community, while the former came from infections acquired in hospitals. Unless multiple blood culture bottles are used to rule out infection versus contamination [67], the other possibility may be related to the risk of contamination of blood culture from the environment by *Klebsiella* species from hospital-acquired infections. Some microbiology laboratories in developing countries use single bottles for pediatric blood cultures due to supply shortages [68].

Every individual, at any stage of life, is susceptible to AMR. Individuals who are undergoing medical treatment or have compromised immune systems are frequently more vulnerable to infection [69]. According to global estimates for 2019, children bear a disproportionate share of the burden of death: of the 1.27 million deaths directly attributable to AMR, 254,000 occurred among those under the age of five, accounting for approximately 20% of all deaths. This is the equivalent of one child passing away almost every two minutes. More than 99 percent of those 254,000 kids are from low- and middle-income countries, and more than half of them pass away in their first month of life. Stated differently, 900 children in high-income countries (HICs) and nearly 253,000 in low- and middle-income countries (LMICs) died directly from AMR [70]. Our study also encompassed a subgroup analysis based on the age of the study subjects, which revealed that the lower age group (children and neonates) had a higher prevalence of MDR *Klebsiella* species (97% (91–100%) vs 96% (92–100%)). Several facets of this growing problem are unique to children. Without immediate action, we are at risk of entering a post-antibiotic era in which common infections could once again be fatal in such a rapidly changing environment. This is especially true for children and babies less than a week old, as their gastrointestinal tracts contain populations of MDR bacteria, most likely because of exposure to mother and environmental bacteria during and right after delivery [71].

In our review, a higher MDR *Klebsiella* species were identified from HAI 97% (97–100%). This might be because staying in a hospital or other healthcare facility increases the risk of contracting an antibiotic-resistant infection. Antibiotic exposure is frequent for patients in these facilities, and they are often prone to a lot of hands-on care which may expose them to resistant bacteria from either medical equipment or health professionals. Furthermore, hospitals are more likely than communities to harbour the majority of resistant bacteria [72].

In general, our study has the following limitations. One of the study’s limitations is that it only looked at disc diffusion; it did not find a study which used minimum inhibitory concentration (MIC) or molecular techniques. It only used patient specimens, focused on studies conducted between 2018 and 2022, and failed to determine whether certain specimens, such as urine, faeces, sputum, and discharges, were from infection or colonization. Our study considered the report of MDR *Klebsiella* spp. as one of the eligibility criteria but not the definition of MDR because the studies used different definitions and some included simply the MDR value.

## Conclusion

Our study revealed that the prevalence of MDR *Klebsiella* species was high in Ethiopia. The subgroup analyses elaborated on the proportion of MDR *Klebsiella* spp. that was different between regional states, types of specimens, study participants’ ages, sources and types of infection. Therefore, integrated action should be taken to reduce the rates of multi-drug-resistant *Klebsiella* to regional states, age, and focus on clinical features of patients. Standard precautions should be applied to reduce the transmission of MDR *Klebsiella* spp in hospital and out of the hospital.

## Supporting information

S1 Checklist Human participants research checklist. (DOCX)

S1 Table PRISMA 2020 checklist. (DOCX)

S2 Table The quality score of each study based on Joanna Briggs Institute (JBI). (DOCX)

## References

[1] Martin R. M., Bachman M. A. Colonization, infection, and the accessory genome of Klebsiella pneumoniae. Front Cell Infect Microbiol. 2018; 8: 4. doi: 10.3389/fcimb.2018.00004 29404282 PMC5786545

[2] Britannica T. Editors of Encyclopaedia. "klebsiella." Encyclopedia Britannica, September 23, 2023. https://www.britannica.com/science/Klebsiella.

[3] Wyres KL, Lam MM, Holt K. Population genomics of Klebsiella pneumoniae. Nat Rev Microbiol. 2020; 18 (6): 344–35. doi: 10.1038/s41579-019-0315-1 32055025

[4] Chen L, Kreiswirth BN. Kreiswirth, Convergence of carbapenem-resistance and hypervirulence in Klebsiella pneumoniae. Lancet Infect Dis, 2018. 18 (1): 2–3. doi: 10.1016/S1473-3099(17)30517-0 28864028

[5] Gu D, Dong N, Zheng Z, Lin D, Huang M, Wang L et al. A fatal outbreak of ST11 carbapenem-resistant hypervirulent Klebsiella pneumoniae in a Chinese hospital: a molecular epidemiological study. Lancet Infect Dis. 2018; 18(1): 37–46. doi: 10.1016/S1473-3099(17)30489-9 28864030

[6] Russo TA, Marr CM. Hypervirulent Klebsiella pneumoniae. Clin Microbiol Rev. 2019; 32(3): 10–128 doi: 10.1128/cmr.00001-19 31092506 PMC6589860

[7] Dong N, Yang X, Zhang R, Chan EW, Chen S. Tracking microevolution events among ST11 carbapenemases-producing hypervirulent Klebsiella pneumoniae outbreak strains. Emerg Microbes Infect. 2018; 7(1): 1–8. doi: 10.1038/s41426-018-0146-6 30100608 PMC6087712

[8] Tang M, Kong X, Hao J, Liu J. Epidemiological characteristics and formation mechanisms of multidrug-resistant hypervirulent Klebsiella pneumoniae. Front Microbiol. 2020; 11: .581543. doi: 10.3389/fmicb.2020.581543 33329444 PMC7714786

[9] Marr CM, Russo T. Hypervirulent Klebsiella pneumoniae: a new public health threat. Expert Rev Anti Infect Ther. 2019; 17 (2): 71–73. doi: 10.1080/14787210.2019.1555470 30501374 PMC6349525

[10] World Health Organization (WHO). Prioritization of pathogens to guide discovery, research, and development of new antibiotics for drug-resistant bacterial infections, including tuberculosis,2017. https://www.who.int/publications/i/item/WHO-EMP-IAU-2017.12.

[11] Awoke T, Teka B, Seman A, Sebre S, Yeshitela B, Aseffa A, et al. High prevalence of multidrug-resistant Klebsiella pneumoniae in a tertiary care hospital in Ethiopia. Antibiotics (Basel). 2021; 10 (8): 1007. doi: 10.3390/antibiotics10081007 34439057 PMC8388856

[12] Khan H, Malik A, Gaur PK, Rastogi S, Lata K. Antibiotic Resistance: A Universal Issue of Concern. JPPCM, 2022; 8 (1): 4–7. doi: 10.5530/jppcm.2022.1.2

[13] Meatherall BL, Gregson D, Ross T, Pitout JD, Laupland KB. Incidence, risk factors, and outcomes of Klebsiella pneumoniae bacteremia. Am J Med 2009;122 (9): 866–873. doi: 10.1016/j.amjmed.2009.03.034 19699383

[14] Collingwood A, Blostein F, Seekatz AM, Wobus CE, Woods RJ, Foxman B, et al. Epidemiological and Microbiome Associations Between Klebsiella pneumoniae and Vancomycin-Resistant Enterococcus Colonization in Intensive Care Unit Patients. Open Forum Infect Dis, 2020, 7(1): 012. doi: 10.1093/ofid/ofaa012 32010736 PMC6984673

[15] Martin RM, Cao J, Wu W, Zhao L, Manthei DM, Pirani A, et al. Identification of pathogenicity-associated loci in Klebsiella pneumoniae from hospitalized patients. mSystems. 2018; 3 (3):10–128. doi: 10.1128/mSystems.00015-18 29963640 PMC6020474

[16] Li Y, Kumar S, Zhang L, Wu H. Klebsiella pneumonia and Its Antibiotic Resistance: A Bibliometric Analysis. Biomed Res Int. 2022 Jun 6: 1668789. doi: 10.1155/2022/1668789 35707374 PMC9192197

[17] Moher D, Shamseer L, Clarke M, Ghersi D, Liberati A, Petticrew Met al., Preferred reporting items for systematic review and meta-analysis protocols (PRISMA-P) 2015 statement. Syst Rev, 2015. 4(1): 1–9. doi: 10.1186/2046-4053-4-1 25554246 PMC4320440

[18] Munn Z, Moola S, Lisy K, Riitano D, Tufanaru C. Methodological guidance for systematic reviews of observational epidemiological studies reporting prevalence and cumulative incidence data. JBI Evid Implement, 2015. 13(3): 147–153. doi: 10.1097/XEB.0000000000000054 26317388

[19] Moola S, Munn Z, Sears K, Sfetcu R, Currie M, Lisy Ket al., Conducting systematic reviews of association (aetiology): The Joanna Briggs Institute’s approach. JBI Evid Implement, 2015. 13(3): 163–169. doi: 10.1097/XEB.0000000000000064 26262566

[20] Admas A, Gelaw B, Belay Tessema, Worku A, Melese A. Proportion of bacterial isolates, their antimicrobial susceptibility profile and factors associated with puerperal sepsis among post-partum/aborted women at a referral Hospital in Bahir Dar, Northwest Ethiopia. Antimicrob Resist Infect Control, 2020. 9(1): 1–10. doi: 10.1186/s13756-019-0676-2 31956403 PMC6958633

[21] Adugna B, Sharew B, Jemal M., Bacterial Profile, Antimicrobial Susceptibility Pattern, and Associated Factors of Community-and Hospital-Acquired Urinary Tract Infection at Dessie Referral Hospital, Dessie, Northeast Ethiopia. Int J Microbiol, 2021. 2021 1–14. doi: 10.1155/2021/5553356 34589128 PMC8476241

[22] Ali IE, Gebrecherkos T, Gizachew M, Menberu MA. Asymptomatic bacteriuria and antimicrobial susceptibility pattern of the isolates among pregnant women attending Dessie referral hospital, Northeast Ethiopia: A hospital-based cross-sectional study. Turk J Urol, 2018. 44(3): 251. doi: 10.5152/tud.2018.07741 29733799 PMC5937645

[23] Ameshe A, Engda T, Gizachew M. Prevalence, antibiotic susceptibility patterns, including ESBL production and associated risk factors of Klebsiella species among UTI suspected patients at Bahir-dar town, Northwest Ethiopia. doi: 10.21203/rs.3.rs-24907/v1 PMC8960036 35355926

[24] Assefa M, Tigabu A, Belachew T, Tessema B. Bacterial profile, antimicrobial susceptibility patterns, and associated factors of community-acquired pneumonia among adult patients in Gondar, Northwest Ethiopia: A cross-sectional study. PLoS One, 2022. 17(2): e0262956. doi: 10.1371/journal.pone.0262956 35104293 PMC8806065

[25] Belete Y, Asrat D, Woldeamanuel Y, Yihenew G, Gize A. Bacterial profile and antibiotic susceptibility pattern of urinary tract infection among children attending Felege Hiwot Referral Hospital, Bahir Dar, Northwest Ethiopia. Infect Drug Resist, 2019: 3575–3583. doi: 10.2147/IDR.S217574 31819542 PMC6874112

[26] Belyhun Y, Moges F, Endris M, Asmare B, Amare B, Bekele D, et al., Ocular bacterial infections and antibiotic resistance patterns in patients attending Gondar Teaching Hospital, Northwest Ethiopia. BMC Res Notes, 2018. 11: 1–7. doi: 10.1186/s13104-018-3705-y 30119696 PMC6098601

[27] Girma A, Aemiro A. The Bacterial Profile and Antimicrobial Susceptibility Patterns of Urinary Tract Infection Patients at Pawe General Hospital, Northwest Ethiopia. Scientifica (Cairo), 2022. doi: 10.1155/2022/3085950 35509515 PMC9061053

[28] Haile Z, Mengist HM, Dilnessa T. Bacterial isolates, their antimicrobial susceptibility pattern, and associated factors of external ocular infections among patients attending eye clinic at Debre Markos Comprehensive Specialized Hospital, Northwest Ethiopia. PLoS One, 2022. 17(11): e0277230. doi: 10.1371/journal.pone.0277230 36327266 PMC9632921

[29] Molla R, Tiruneh M, Abebe W, Moges F. Bacterial profile and antimicrobial susceptibility patterns in chronic suppurative otitis media at the University of Gondar Comprehensive Specialized Hospital, Northwest Ethiopia. BMC Res Notes, 2019. 12: 1–6. doi: 10.1186/s13104-019-4452-4 31307545 PMC6631645

[30] Oumer O, Metaferia Y, Gebretsadik D. Bacterial uropathogens, their associated factors, and antimicrobial susceptibility pattern among adult diabetic patients in two health centres at Kombolcha town, Northeastern Ethiopia. SAGE Open Med, 2022. 10: doi: 10.1177/20503121221139149 36457843 PMC9706039

[31] Sahle Z, Engidaye G, Shenkute D, Metaferia Y, Shibabaw A. High Prevalence of Multi-Drug Resistance and Extended-Spectrum Beta-Lactamase-Producing Enterobacteriaceae Among Hospitalized Patients Presumptive for Bacterial Infection at Debre Berhan Comprehensive Specialized Hospital, Ethiopia. Infection and Drug Resistance, 2022:. 2639–2656. doi: 10.2147/IDR.S363988 35642212 PMC9148578

[32] Shenkute D, Legese MH, Yitayew B, Mitiku A, Engidaye G, Gebremichael S et al. High Magnitude of Fecal Carriage of Extended-Spectrum Beta-Lactamase-Producing Enterobacteriaceae at Debre Berhan Comprehensive Specialized Hospital, Ethiopia. Infect Drug Resist, 2022: 15: 2445–2458. doi: 10.2147/IDR.S356807 35586558 PMC9109895

[33] Tigabu A, Ferede W, Belay G, Gelaw B. Prevalence of asymptomatic bacteriuria and antibiotic susceptibility patterns of bacterial isolates among cancer patients and healthy blood donors at the University of Gondar Specialized Hospital. Int J Microbiol, 2020. doi: 10.1155/2020/3091564 32377201 PMC7183528

[34] Tilahun M, Gebretsadik D, Seid A, Gedefie A, Belete MA, Tesfaye Met al., Bacteriology of community-acquired pneumonia, antimicrobial susceptibility pattern and associated risk factors among HIV patients, Northeast Ethiopia: cross-sectional study. SAGE Open Med, 2023. 11: doi: 10.1177/20503121221145569 36632083 PMC9827525

[35] Worku M, Belay G, Tigabu A., Bacterial profile and antimicrobial susceptibility patterns in cancer patients. PLoS One, 2022. 17(4): e0266919. doi: 10.1371/journal.pone.0266919 35427384 PMC9012398

[36] Yasin J, Ayalew G, Dagnaw M, Shiferaw G, Mekonnen F. Vulvovaginitis prevalence among women in Gondar, Northwest Ethiopia: Special emphasis on aerobic vaginitis causing bacterial profile, antimicrobial susceptibility pattern, and associated factors. Infect Drug Resist, 2021;14:4567–4580. doi: 10.2147/IDR.S337205 34754204 PMC8572045

[37] Zenebe Y, Molla T, Beza L, Mekonnen D., Bacterial profile and antimicrobial susceptibility pattern of neonatal sepsis in Felege-Hiwot Referral Hospital, Bahir Dar, northwest Ethiopia: A cross-sectional study design. Ethiop J Health Dev, 2021. 35(1).18–28.

[38] Fenta A, Dagnew M, Eshetie S, Belachew T. Bacterial profile, antibiotic susceptibility pattern and associated risk factors of urinary tract infection among clinically suspected children attending at Felege-Hiwot comprehensive and specialized hospital, Northwest Ethiopia. A prospective study. BMC Infect Dis, 2020. 20: 673. doi: 10.1186/s12879-020-05402-y 32938424 PMC7493977

[39] Bizuayehu H, Bitew A, Abdeta A, Ebrahim S. Catheter-associated urinary tract infections in adult intensive care units at a selected tertiary hospital, Addis Ababa, Ethiopia. PloS One, 2022. 17(3): e0265102. doi: 10.1371/journal.pone.0265102 35316286 PMC8939826

[40] Bizuwork K, Alemayehu H, Medhin G, Amogne W, Eguale T. Asymptomatic bacteriuria among pregnant women in Addis Ababa, Ethiopia: prevalence, causal agents, and their antimicrobial susceptibility. Int J Microbiol, 2021. doi: 10.1155/2021/8418043 34335781 PMC8313335

[41] Merga Duffa Y, Terfa Kitila K, Mamuye Gebretsadik D, Bitew A. Prevalence and Antimicrobial Susceptibility of Bacterial Uropathogens Isolated from Pediatric Patients at Yekatit 12 Hospital Medical College, Addis Ababa, Ethiopia. Int J Microbiol, 2018. doi: 10.1155/2018/8492309 30386381 PMC6189692

[42] Sherif M, Abera D, Desta K. Prevalence and antibiotic susceptibility pattern of bacteria from sepsis suspected neonates at St. Paul Hospital Millennium Medical College, Addis Ababa, Ethiopia. 2022. doi: 10.21203/rs.3.rs-2276441/v1 PMC10656775 37980512

[43] Wabe YA, Reda DY, Abreham ET, Gobene DB, Ali MM. Prevalence of asymptomatic bacteriuria, associated factors and antimicrobial susceptibility profile of bacteria among pregnant women attending Saint Paul’s Hospital Millennium Medical College, Addis Ababa, Ethiopia. Ther Clin Risk Manag, 2020;16 923–932. doi: 10.2147/TCRM.S267101 33061397 PMC7532909

[44] Yenehun W, Belete A, Erku A, Prevalence of bacterial urinary tract infection and antimicrobial susceptibility patterns among diabetes mellitus patients attending Zewditu Memorial Hospital, Addis Ababa, Ethiopia. Infect Drug Resist, 2021;:14: 1441–1454. doi: 10.2147/IDR.S298176 33883912 PMC8055279

[45] Woreta AN, Kebede HB, Tilahun Y, Teklegiorgis SG, Abegaz WE. Antibiotic susceptibility pattern and bacterial spectrum among patients with external eye infections at Menelik II Referral Hospital in Addis Ababa, Ethiopia. Infect Drug Resist, 2022; 15:: 765–779. doi: 10.2147/IDR.S352098 35264860 PMC8901190

[46] Ameya G, Weldemedhin T, Tsalla T, Gebremeskel F. Antimicrobial Susceptibility Pattern and Associated Factors of Pediatric Septicemia in Southern Ethiopia. Infect Drug Resist 2020.;13: 3895–3905.doi: 10.2147/IDR.S278293 33154655 PMC7608546

[47] Birru M, Woldemariam M, Manilal A, Aklilu A, Tsalla T, Mitiku A, et al., Bacterial profile, antimicrobial susceptibility patterns, and associated factors among bloodstream infection suspected patients attending Arba Minch General Hospital, Ethiopia. Sci Rep, 2021; 11(1): 15882. doi: 10.1038/s41598-021-95314-x 34354138 PMC8342484

[48] Haile Hantalo A, Haile Taassaw K, Solomon Bisetegen F, Woldeamanuel Mulate Y. Isolation and Antibiotic Susceptibility Pattern of Bacterial Uropathogens and Associated Factors Among Adult People Living with HIV/AIDS Attending the HIV Center at Wolaita Sodo University Teaching Referral Hospital, South Ethiopia. HIV AIDS (Auckl) 2020. 12: 799–808. doi: 10.2147/HIV.S244619 33273865 PMC7708265

[49] Mitiku A, Aklilu A, Tsalla T, Woldemariam M, Manilal A, Biru M., Magnitude and antimicrobial susceptibility profiles of Gram-Negative bacterial isolates among patients suspected of urinary tract infections in Arba Minch General Hospital, southern Ethiopia. PLoS One, 2022. 17(12): e0279887. doi: 10.1371/journal.pone.0279887 36584225 PMC9803306

[50] Oumer Y, Regasa Dadi B, Seid M, Biresaw G, Manilal A. Catheter-associated urinary tract infection: Incidence, associated factors and drug resistance patterns of bacterial isolates in southern Ethiopia. Infect Drug Resist, 2021;142883–2894. doi: 10.2147/IDR.S311229 34335034 PMC8318706

[51] Alemayehu T, Tadesse E, Ayalew S, Nigusse B, Yeshitila B, Amsalu A. et al., High burden of nosocomial infections caused by multi-drug resistant pathogen in pediatric patients at Hawassa University Comprehensive Specialized Hospital Ethiop Med J, 2019, Suppl. 1

[52] Gebre AB, Begashaw TA, Ormago MD., Bacterial profile and drug susceptibility among adult patients with community-acquired lower respiratory tract infection at tertiary hospital, Southern Ethiopia. BMC Infect Dis, 2021. 21(1): 440. doi: 10.1186/s12879-021-06151-2 33985445 PMC8120775

[53] Mechal T, Hussen S, Desta M. Bacterial Profile, Antibiotic Susceptibility Pattern, and Associated Factors Among Patients Attending Adult OPD at Hawassa University Comprehensive Specialized Hospital, Hawassa, Ethiopia. Infect Drug Resist, 2021. 14: 99–110. doi: 10.2147/IDR.S287374 33469325 PMC7813457

[54] Ejerssa AW, Gadisa DA, Orjino TA. Prevalence of bacterial uropathogens and their antimicrobial susceptibility patterns among pregnant women in Eastern Ethiopia: hospital-based cross-sectional study. BMC Womens Health, 2021. 21(1): 291. doi: 10.1186/s12905-021-01439-6 34364376 PMC8348837

[55] Tolera M, Abate D, Dheresa M, Marami D. Bacterial nosocomial infections and antimicrobial susceptibility pattern among patients admitted at Hiwot Fana Specialized University Hospital, Eastern Ethiopia. Adv Med, 2018. doi: 10.1155/2018/2127814 30631777 PMC6305041

[56] Marami D, Balakrishnan S, Seyoum B. Prevalence, Antimicrobial Susceptibility Pattern of Bacterial Isolates, and Associated Factors of Urinary Tract Infections among HIV-Positive Patients at Hiwot Fana Specialized University Hospital, Eastern Ethiopia. Can J Infect Dis Med Microbiol, 2019:.: 6780354. doi: 10.1155/2019/6780354 30881531 PMC6381576

[57] Adhanom G, Gebreegziabiher D, Weldu Y, Gebreyesus Wasihun A, Araya T, Legese H. et al., Species, risk factors, and antimicrobial susceptibility profiles of bacterial isolates from HIV-infected patients suspected to have pneumonia in Mekelle zone, Tigray, northern Ethiopia. Biomed Res Int, 2019. doi: 10.1155/2019/8768439 31192259 PMC6525850

[58] Gebremariam G, Legese H, Woldu Y, Araya T, Hagos K, Gebreyesus W. Bacteriological profile, risk factors and antimicrobial susceptibility patterns of symptomatic urinary tract infection among students of Mekelle University, northern Ethiopia. BMC Infect Dis, 2019. 19: 950. doi: 10.1186/s12879-019-4610-2 31703645 PMC6842233

[59] Negussie A, Worku G, Beyene E. Bacterial identification and drug susceptibility pattern of urinary tract infection in pregnant women at Karamara Hospital Jigjiga, Eastern Ethiopia. Afr J Bacteriol Res. 2018; 10(2):15–22 doi: 10.5897/JBR2017.0255

[60] Centres for Disease Control and Prevention (CDC), 2021. Antibiotic Resistance & Patient Safety Portal. https://www.cdc.gov/drugresistance/index.html.

[61] Mohd Asri, Ahmad S, Mohamud R, Mohd Hanafi N, Mohd Zaidi NF, Irekeola AA, et al., Global prevalence of nosocomial multidrug-resistant Klebsiella pneumoniae: a systematic review and meta-analysis. Antibiotics (Basel), 2021; 10(12): 1508. doi: 10.3390/antibiotics10121508 34943720 PMC8698758

[62] Shyaula M, Khadka C, Dawadi P, Banjara MR. Systematic Review and Meta-analysis on Extended-Spectrum β-lactamases Producing Klebsiella pneumoniae in Nepal. Microbiol Insights, 2023. 16:1–13 doi: 10.1177/11786361221145179 36655025 PMC9841864

[63] Odari R, Dawadi P. Prevalence of multidrug-resistant Klebsiella pneumoniae clinical isolates in Nepal. J Trop Med 2022. doi: 10.1155/2022/5309350 35242192 PMC8888090

[64] Tweldemedhin M, Muthupandian S, Gebremeskel TK, Mehari K, Abay GK, Teklu TG, et al. Multidrug resistance from a one-health perspective in Ethiopia: A systematic review and meta-analysis of literature (2015–2020). One Health, 2022. 14: 100390. doi: 10.1016/j.onehlt.2022.100390 35686143 PMC9171526

[65] Centers for Disease Control and Prevention (CDC), Antimicrobial resistance. Where Resistance Spreads: Where Resistance Spreads: Water, Soil, & the Environment, 2021. https://www.cdc.gov/drugresistance/environment.html.

[66] World Health Organization(WHO). Antimicrobial resistance, 23, November, 2023. https://www.who.int/news-room/fact-sheets/detail/antimicrobial-resistance.

[67] Lamy B, Dargère S, Arendrup MC, Parienti JJ, Tattevin P. How to optimize the use of blood cultures for the diagnosis of bloodstream infections? A state-of-the art. Front Microbiol, 2016; 7:697. doi: 10.3389/fmicb.2016.00697 27242721 PMC4863885

[68] Ombelet S, Barbé B, Affolabi D, Ronat JB, Lompo P, Lunguya O, et al. Best Practices of Blood Cultures in Low- and Middle-Income Countries. Front Med (Lausanne),2019 6: 131. doi: 10.3389/fmed.2019.00131 31275940 PMC6591475

[69] Centers for Disease Control and Prevention (CDC) Antimicrobial prescription and use. Antimicrobial Resistance Questions and Answers/Antibiotic Use, 2022. https://www.cdc.gov/antibiotic-use/q-a.html.

[70] UNICEF. The urgent threat of drug-resistant infections protecting children worldwide. https://www.unicef.org/documents/amr-urgent-threat-drug-resistant-infections.

[71] Romandini A, Pani A, Schenardi PA, Pattarino GA, De Giacomo C, Scaglione F. Antibiotic resistance in pediatric infections: global emerging threats, predicting the near future. Antibiotics (Basel). 2021; 10 (4):393. doi: 10.3390/antibiotics10040393 33917430 PMC8067449

[72] Centers for Disease Control and Prevention (CDC). Healthcare-Associated Infections (HAIs). Antibiotic Resistant Germs in Hospitals, 2019. https://www.cdc.gov/hai/patientsafety/ar-hospitals.html.

