# Supplementary material for: Prevalence of phenotypic multi-drug resistant Klebsiella species recovered from different human specimens in Ethiopia: A systematic review and meta-analysis
Source: PLoS One. 2024 Feb 9;19(2):e0297407. doi: 10.1371/journal.pone.0297407 (PMC10857728; doi:10.1371/journal.pone.0297407)
Supplement: S2 Table — (DOCX) [file pone.0297407.s003.docx]

S2 Table: the quality score of each study based on Joanna Briggs Institute (JBI) f

| SN | Author | Was the sample frame appropriate to address the target population? | Were study participants sampled in an appropriate way? | Was the sample size adequate? | Were the study subjects and the setting described in detail? | Was the data analysis conducted with sufficient coverage of the identified sample? | Were valid methods used for the identification of the condition? | Was the condition measured in a standard, reliable way for all participants? | Was there appropriate statistical analysis? | Was the response rate adequate, and if not, was the low response rate managed appropriately? | Q score | |
| --- | --- | --- | --- | --- | --- | --- | --- | --- | --- | --- | --- | --- |
|  |  |  |  |  |  |  |  |  |  |  | No | % |
|  | Duffa *et al.* [1] | 1 | 1 | 1 | 1 | 1 | 1 | 1 | 1 | 0 | 8 | 88.8 |
|  | Sherif [3] | 1 | 1 | 1 | 1 | 1 | 1 | 1 | 1 | 0 | 8 | 88.8 |
|  | Ejerssa *et al.* [4] | 1 | 0 | 1 | 1 | 1 | 1 | 1 | 1 | 1 | 8 | 88.8 |
|  | Molla *et al.*[5] | 1 | 0 | 0 | 1 | 1 | 1 | 1 | 1 | 0 | 6 | 66.6 |
|  | Birru *et al*. [8] | 1 | 1 | 1 | 1 | 1 | 1 | 1 | 1 | 1 | 9 | 100 |
|  | Yenehun *et al.*[10] | 1 | 1 | 1 | 1 | 1 | 1 | 1 | 1 | 0 | 8 | 88.8 |
|  | Bizuwork *et al.*, [11] | 1 | 1 | 1 | 1 | 1 | 1 | 1 | 1 | 0 | 8 | 88.8 |
|  | Bizuayehu *et al.*[13] | 1 | 0 | 1 | 1 | 1 | 1 | 1 | 1 | 0 | 7 | 77.7 |
|  | Mitiku *et al.*[14] | 1 | 1 | 1 | 1 | 1 | 1 | 1 | 1 | 1 | 9 | 100 |
|  | Tigabu *et al.*[17] | 1 | 1 | 1 | 1 | 1 | 1 | 1 | 1 | 0 | 8 | 88.8 |
|  | Ali *et al.*[18] | 1 | 1 | 1 | 1 | 1 | 1 | 1 | 1 | 0 | 8 | 88.8 |
|  | Adugna[19] | 1 | 1 | 1 | 1 | 1 | 1 | 1 | 1 | 1 | 9 | 100 |
|  | Wabe*et al.*[20] | 1 | 1 | 1 | 1 | 1 | 1 | 1 | 1 | 1 | 9 | 100 |
|  | Belete*et al.* [21] | 1 | 0 | 1 | 1 | 1 | 1 | 1 | 1 | 1 | 8 | 88.8 |
|  | Molla *et al.* [22] | 1 | 0 | 1 | 1 | 1 | 1 | 1 | 1 | 0 | 7 | 77.7 |
|  | Fenta *et al.* [23] | 1 | 0 | 1 | 1 | 1 | 1 | 1 | 1 | 0 | 7 | 77.7 |
|  | Tolera*et al.* [24] | 1 | 0 | 1 | 1 | 1 | 1 | 1 | 1 | 1 | 8 | 88.8 |
|  | Ameshe [25] | 1 | 1 | 1 | 1 | 1 | 1 | 1 | 1 | 0 | 8 | 88.8 |
|  | Adhanom *et al.* [27] | 1 | 0 | 1 | 1 | 1 | 1 | 1 | 1 | 0 | 7 | 77.7 |
|  | Oumer *et al.* [28] | 1 | 1 | 1 | 1 | 1 | 1 | 1 | 1 | 1 | 9 | 100 |
|  | Mechal *et al.* [29] | 1 | 1 | 1 | 1 | 1 | 1 | 1 | 1 | 1 | 9 | 100 |
|  | Marami [31] | 1 | 1 | 1 | 1 | 1 | 1 | 1 | 1 | 1 | 9 | 100 |
|  | Assefa *et al.* [34] | 1 | 1 | 1 | 1 | 1 | 1 | 1 | 1 | 0 | 8 | 88.8 |
|  | Yasin *et al.* [35] | 1 | 0 | 1 | 1 | 1 | 1 | 1 | 1 | 1 | 8 | 88.8 |
|  | Oumer *et al.* [37] | 1 | 0 | 1 | 1 | 1 | 1 | 1 | 1 | 0 | 7 | 77.7 |
|  | Ameya *et al.* [38] | 1 | 1 | 1 | 1 | 1 | 1 | 1 | 1 | 1 | 9 | 100 |
|  | Tilahun *et al.* [40] | 1 | 1 | 1 | 1 | 1 | 1 | 1 | 1 | 0 | 8 | 88.8 |
|  | Girma [41] | 1 | 0 | 0 | 1 | 1 | 1 | 1 | 1 | 0 | 6 | 66.6 |
|  | Gebremariam *et al.* [43] | 1 | 0 | 1 | 1 | 1 | 1 | 1 | 1 | 1 | 8 | 88.8 |
|  | Hantalo *et al*. [36] | 1 | 1 | 1 | 1 | 1 | 1 | 1 | 1 | 1 | 9 | 100 |
|  | Belyhun *et al.* [15] | 0 | 0 | 1 | 0 | 1 | 1 | 1 | 1 | 0 | 5 | 55.5 |
|  | Woreta *et al.* [45] | 1 | 1 | 1 | 1 | 1 | 1 | 1 | 1 | 0 | 8 | 88.8 |
|  | Alemayehu *et al*. [46] | 1 | 1 | 1 | 1 | 1 | 1 | 1 | 1 | 0 | 8 | 88.8 |
|  | Gebre [47] | 1 | 1 | 1 | 1 | 1 | 1 | 1 | 1 | 1 | 9 | 100 |
|  | Haile *et al*. [48] | 1 | 0 | 1 | 1 | 1 | 1 | 1 | 1 | 1 | 8 | 88.8 |
|  | Negussie *et al*. [49] | 1 | 0 | 0 | 1 | 1 | 1 | 1 | 1 | 1 | 7 | 77.7 |
|  | Worku & Tigabu [50] | 1 | 0 | 1 | 1 | 1 | 1 | 1 | 1 | 0 | 7 | 77.7 |
|  | Admas *et al*. [51] | 1 | 0 | 1 | 1 | 1 | 1 | 1 | 1 | 1 | 8 | 88.8 |
|  | Sahle *et al* [52] | 1 | 0 | 1 | 1 | 1 | 1 | 1 | 1 | 0 | 7 | 77.7 |
|  | Shenkute *et al*. [53] | 1 | 0 | 1 | 1 | 1 | 1 | 1 | 1 | 0 | 7 | 77.7 |

These questions can be Graded with ‘1’ for yes and ‘0’ for no, unclear, or not applicable.

Answers: ‘1’ for Yes and ‘0’ for No, Unclear or Not/Applicable
