# Supplementary material for: Prevalence of phenotypic multi-drug resistant Klebsiella species recovered from different human specimens in Ethiopia: A systematic review and meta-analysis
Source: PLoS One. 2024 Feb 9;19(2):e0297407. doi: 10.1371/journal.pone.0297407 (PMC10857728; doi:10.1371/journal.pone.0297407)
Supplement: Attachment — Submitted filename: Point by point response to reviewer.docx [file pone.0297407.s004.docx]

**Point by point response to reviewer**

Dear editor and reviewer we want to thank you about the constructive comments. The manuscript is now improved a lot much improved than before and we are ready to address if further revision is need because it helps for our manuscript. The revised part is shaded with yellow color. Here we give a point-by-point response for the given comments.

**Response to Editor**

**Comment 1:**Generally, while the objective of this study is interesting, the reporting is insufficient (for example, there are major concerns with respect to the study outcome definition) and there are major concerns regarding the data analysis and discussion write-up. The conclusion drawn from the study is simply descriptive and does not provide recommendations for policy makers on how to cope up with the alarmingly high MDR pooled prevalence.

**Response**: Thank you very much for the constructive comment and the manuscript is now much improved and modified based on the comment.

**Comment 2**: The authors are totally unclear regarding the outcome.

**Response**: we amended the outcome of the study.

**Comment 3**: The abstract and introduction leave the reader in the dark with regard to the exact study outcome.

**Response**: accepted and modified accordingly

**Comment 4:** In the methods section the outcome is then reported as MDR Klebsiella species, but without further detail. If the authors focused on infection, they should clearly explain why they included studies on carriage also (for example, see Ali IE et al. 2018, Tigabu A et al. 2020, Bizuwork K et al. 2021, Wabe et al. 2020 and Gebremariam G et al. 2019 on asymptomatic UTI carriage).

**Response**: we tried to modify the method section based on the comment and we included about the infection and colonization in the limitation section. We modified the title to different human specimen.

**Comment**: Additionally, the authors do not explain the criteria for infection or provide any outcome definitions – individual study outcomes are also not included in the table.

**Response**: accepted and modified. We included the outcome of the study included on the table.

**Comment**: There are very strong concerns regarding the pooling of different specimen types (blood, urine, ear discharge, eye swabs, body fluids etc..) and populations (neonates, adults, HIV patients, pregnant women, cancer patients, etc.) and, of course this leads to huge discrepancies in prevalence.

**Response**: we accepted the comment and conducted a sub-group analysis based on the study subject’s age and others.

**Comment**: There are several grammatical errors throughout the manuscript that need corrections.

**Response**: we accepted and the we tried to modify the senior author edited the manuscript for the grammatical error and if there is any assistance for language editing from you we kindly request the journal for this service as we are from second language speakers.

**Comment**: Have you considered the definitions each authors used to declare an isolate as MDR? You didn’t put the definition of MDR in the inclusion criteria either. I have seen some differences in MDR definitions among included studies and this may raise doubt in the credibility of the findings of this meta-analysis.

**Response**: We use the report of the authors MDR Klebsiella as eligibility criteria not the definition. Different authors used different operational definition and some did not indicated which definition is used, but we put it this one as a limitation of our study.

**Comment**: - The inclusion of a single group of study (only 1 study from Somalia) is inappropriate. The authors should perform sub-group analysis for groups composed of 2 or more studies.

**Response**: the sub-group analysis is conducted for all regions unfortunately; we identified a single study from Somali. Can we excluded for being a single study from all meta-analysis? Or omit from meta analysis?

**Comment**: Search strategies: why did you choose these specific databases? Some of the reported databases have very high overlaps.

**Response**: just we have to increase the chance of identifying the potential articles from multiple sources.

**Comment**: Please include all search strategies in detail in the supplement in line with PRISMA recommendations (database, date searched, key words, number of articles retrieved etc).

**Response**: Included as additional file in both the attachment and manuscript.

**Comment**: Please include the university repositories used and the Google search line as a supplement.

**Response**: accepted and included.

**Comment**: Eligibility criteria: human specimens of what material? It seems you included any material and made no distinction between infection and carriage.

**Response**: We included all studies taken from human specimen. Whether it is infection and carriage and we acknowledge in the limitation section. We modified the title as from different human specimen.

**Comment**: Quality assessment: Did you exclude all other studies with a score <50%? (It seems like you did according to Figure 1). If so, please report them in the supplement. There were no studies with high risk of bias? It would be helpful if the authors can add a legend for the domains of the JBI scoring system.

**Response**: it is included on the supplement file as per comment.

**Comment**: The statistical data analysis used in this study are not enough. The authors claimed that there was high level of heterogeneity however they did not try to assess the cause of the heterogeneity deploying different analysis methods such as sensitivity analysis and meta-regression. Similarly, Egger’s test statistics, Trim, and Fill analysis were not carried out to provide indications of the reliability of the estimate in relation to publication bias. In fact, the authors only used funnel plot to check publication bias. However, this method is not quantitative and leads to inaccurate interpretations when used alone. The absence of these significant statistical analysis tools simply makes this Meta-analysis a descriptive report.

**Response**: we tried to address most of the comments and modified accordingly.

**Comment**: No need to write ‘Disk diffusion’ in Table 1 as all studies used disk diffusion. The authors should mention it in the Study characteristics section.

**Response**: accepted and modified accordingly.

**Comment**: Line 153-157 “However, the meta-analysis result shows the overall pooled prevalence of MDR Klebsiella species in Ethiopia is 72% (63-82%) with a low heterogeneity (I2 = 0.05%) with a significance value of p < 0.01(Figure 2). Since the data was highly heterogeneous (I2 = 95%) subgroup analysis was conducted based on the regional state and specimen type”. These statements are very contradicting and need explanation.

**Response**: Corrected as per comment

**Comment**: I don’t think enough subgroup analysis were carried out in order to assess the source of heterogeneity.

**Response**: corrected as per comment

**Comment**: The Discussion section is poorly written. In fact, it is incorrectly written. The authors must discuss their findings with similar Meta-analysis conducted elsewhere. However, the whole discussion is written by comparing findings of the Meta-analysis with individual studies from different countries and this is totally wrong. Findings of Meta-analysis should be compared with other Meta-analysis reports. This mistake jeopardizes the scientific significance of this paper.

**Response**: corrected as per comment

**Comment**: Was there any limitation in this Meta-analysis? If so, it should be well written at the end of the Discussion section.

**Response**: we included the limitation of the study.

**Comment**: The conclusions lack recommendations so that the outputs of this meta-analysis will be helpful in mitigating prevention and control strategies against MDR Klebsiella spp.

**Response**: modified accordingly

**Response Reviewer 1**

**Comments**: The authors mentioned “Any disagreement that occurred during data extraction between researchers was handled 84 through discussion” What kind of disagreements? It needs explanation.

**Response**: corrected accordingly

**Comment**: One of the questions used in the checklist “Were the study participants sampled appropriately?” Most studies does not show how they calculated the sample size, they just mention the number of study participants. So how the authors addressed this?

**Response**: There are few studies, which omit the study, but even with no response there is possibility of to be included, if it is above 50%. This is just to calculate the Q score.

**Comment**: Did authors used a methods that confirms *Klebsiella* species is pathogenic i.e. not normal flora?

**Response**: that is the difficult most of the time in blood culture and carriage rate, our target is just to determine MDR *Klebsiella* species from clinical specimens. We modified the title to different human specimen.

**Comment**: Did the authors consider MDR definition used by different literatures? If so what definition they used?

**Response**: We use the report of the authors MDR *Klebsiella* as eligibility criteria not the definition. Different authors used different operational definition and some did not indicated which definition is used, but we put it this one as a limitation of our study.

**Comment**: Discussion section: lower and higher prevalence discrepancies between studies should be explained (possible explanations for variations should be given).

**Response**: corrected the discussion

**Comment**: Conclusion section: Any suggestion or recommendations?

**Response**: modified accordingly

**Response to reviewer 2**

**Comment**: The study suggests a high prevalence of multidrug-resistant Klebsiella species in Ethiopia; prevalence varies regionally and according to the sample type analyzed. The language is formal and objective, using appropriate subject-specific terminology. Furthermore, the text is grammatically correct and free from spelling and punctuation errors. The study suggests a high prevalence of multidrug-resistant Klebsiella species in Ethiopia; prevalence varies regionally and according to the sample type analyzed. Technical term abbreviations are explained when first used. Consistent citation and footnote styles are followed, adhering to conventional structure.

**Response**: we have to thank for constructive comments.

**Comment**: The literature search and data collection were conducted appropriately, but the text and the presentation of figures do not correspond.

**Response**: amended as per comment

**Comment**: I understand that this is a review paper, and while the authors may wish to pay homage to the referenced articles, the figures appear cluttered, convoluted and difficult to understand with references to et al.

**Response**: we modified to table

**Comment**: To compare the Ethiopian data with other countries, the authors could produce a single figure, which will be a good addition to the review.

**Response**: we modified the discussion

**Response to reviewer 3**

**Comment**: Title: Phenotypic and species must be added. Prevalence of Phenotypic Multi-drug resistant Klebsiella species recovered from clinical specimens in Ethiopia: A Systematic Review and Meta-analysis.

**Response**: modified according the comment

**Comment**: Introduction: revised line No 44 and 55 at the following positions:

**Response**: revised accordingly

**Comment**: communal flora: commensals flora

**Response**: modified

**Comment**: intestinal tract of humans and animals

**Response**: modified

**Comment**: for infections caused by these problematic species

**Response**: modified

**Comment**: Discussion: Not enough:

**Response**: modified

**Comment**: It is better to highlight the important of the study in the beginning of discussion.

**Response**: modified

**Comment**: More scientific explanation of the results is needed, such as possible causes of MDR occurrence.

**Response**: modified

**Comment**: References No 50, 51 and 52 talked about Klebsiella pneumoniae, so it is not true to mention *Klebsiella* spp there ? And author should mention Klebsiella pneumonia.

**Response**: modified

**Comment**: Study limitation: The author should to discuss the following limitations:

**Response**: included

**Comment**: Absence of multi-drug resistance detection by molecular methods2. The study period covered was short

**Response**: modified
