# Supplementary material for: Prevalence of phenotypic multi-drug resistant Klebsiella species recovered from different human specimens in Ethiopia: A systematic review and meta-analysis
Source: PLoS One. 2024 Feb 9;19(2):e0297407. doi: 10.1371/journal.pone.0297407 (PMC10857728; doi:10.1371/journal.pone.0297407)
Supplement: Attachment — Submitted filename: Point by point Response.docx [file pone.0297407.s005.docx]

**Point-by-Point Response**

**Dear Editor,**

Thank you for your supportive comments to our manuscript that increase its quality. Here we gave a point by point response for the given comments.

**Comment**: The authors tried to modify the manuscript; however, there are still some unaddressed issues in the revision despite being mentioned as modified in the point-by-point response. The newly added subgroup analysis is indeed vital and can now significantly show the probable differences in pooled estimates.

**Response**: thank you for the overall comment and we accept as it is modified our mistake.

**Comment**: Please remove those columns with only 1 study included in the subgroup analysis because such data are once mentioned in your forest plot; for instance, Table 2: Somali regional state.

**Response**: modified as per your comment.

**Comment: “**However, this study revealed bias because of fifteen unpublished papers.” I don’t think this statement is correct as the inclusion of unpublished papers in SRMA cannot be a cause for bias. Please provide elaboration or make an amendment. Please make improvements to the statements under subsection ‘Publication bias’ as it sounds misleading.

**Response**: amended as per your comment

**Comment**: Please remove Fig 5 and all the corresponding statements.

**Response**: modified

**Comment**: The subsection subgroup analysis is enough in your result. Please remove the subsections Subgroup analysis by Regional State, and Subgroup analysis by specimen…. Besides, what was the significance of Table 2 if you mentioned all data in elaborative statements under the subsection? I suppose you should minimize the information in the elaborative statements as the Table is expressive by itself.

**Response**: accept the comment and modify.

**Comment**: Add captions for your Supplementary files.

**Response**: included the caption

**Comment**: I can’t find all search strategies in detail in a supplement file in line with PRISMA recommendations (database, date searched, keywords, number of articles retrieved etc.).

## Response: we included all in the search in a literature search strategy, data extraction and flow diagram.

## Comment: Table 4: Please change the heading of the 2nd column to ‘Study omitted’, remove the word omitting for the rest of the rows, and apply proper citation of the studies inside the table.

**Response**: we accepted and modified accordingly.

**Comment**: Please remove Supporting File 2 (zip file). The individual eligible studies are not required to be placed as a supporting file.

**Response**: Accepted and modified

**Comment**: There are still plenty of typographical errors that need your immediate improvement.

**Response**: we tried to modify as much as we could. We can modify it further if it is indicated.

**Comment**: Discussion 1st paragraph last phrase “antibiosis consumption” should be written as “antibiotic consumption”.

**Response**: modified accordingly.

**Comment**: Figure 1: “Tile/abstract screened (n=81)” Please correct it.

**Response**: Modified
